# Temperature control after successful resuscitation from cardiac arrest in adults: a joint statement from the European Society for Emergency Medicine (EUSEM) and the European Society of Anaesthesiology and Intensive Care (ESAIC)

**DOI:** 10.1097/MEJ.0000000000001106

**Published:** 2023-12-21

**Authors:** Wilhelm Behringer, Bernd W. Böttiger, Daniele G. Biasucci, Athanasios Chalkias, Jim Connolly, Christoph Dodt, Abdo Khoury, Said Laribi, Robert Leach, Giuseppe Ristagno

**Affiliations:** a Department of Emergency Medicine, Medical University Vienna, Vienna General Hospital, Vienna, Austria; b Department of Anesthesiology and Intensive Care Medicine, University Hospital Cologne, Cologne, Germany; c Department of Clinical Science and Translational Medicine, ‘Tor Vergata’ University of Rome, Rome, Italy; d Institute for Translational Medicine and Therapeutics, University of Pennsylvania Perelman School of Medicine, Philadelphia, USA; e Outcomes Research Consortium, Cleveland, Ohio, USA; f Accident and Emergency, Great North Trauma and Emergency Care, Newcastle-upon-Tyne, UK; g Department of Emergency Medicine, München Klinik, Munich, Germany; h Department of Emergency Medicine and Critical Care, Besançon University Hospital, Besançon; i Department of Emergency Medicine, Tours University Hospital, Tours, France; j Department of Emergency Medicine, Centre Hospitalier de Wallonie Picarde, Tournai, Belgium; k Department of Anesthesia, Critical Care and Emergency, Fondazione IRCCS Ca’ Granda Ospedale Maggiore Policlinico, Milan, Italy

## Background

Out-of-hospital cardiac arrest (OHCA) is the third leading cause of death in Europe, with a high burden of disability for patients and their families [1]. When the heart stops, the body and brain cells quickly deplete of oxygen. Without intervention, brain damage occurs rapidly, and death is inevitable. Unfortunately, the prognosis for OHCA patients remains poor, even when return of spontaneous circulation (ROSC) is achieved. Only a few (less than 10%) of OHCA patients can be discharged from the hospital, and only two-thirds of these are discharged with a good neurologic outcome to lead an independent life [1].

Reperfusion injury starts immediately following ROSC. Multiple pathophysiologic cascades lead to reactive astrogliosis and microglia activation and neuronal death by necrosis and apoptosis. This is one of the key component of what has been described as ‘post-resuscitation syndrome’ [2]. Mild hypothermia in the temperature range of 32 to 34°C was shown to mitigate these different pathophysiologic cascades simultaneously, efficiently limiting brain cell damage [3]. Numerous animal studies confirmed the beneficial effect of mild hypothermia [4]. In 2002, two landmark randomized clinical trials (RCT) in patients after cardiac arrest with shockable rhythm showed improved neurological outcomes following treatment with mild hypothermia in the range of 32 to 34°C compared to no temperature control [5,6]. As a result of these studies, in 2005, the European Resuscitation Council (ERC) guidelines recommended the use of mild hypothermia in the range of 32 to 34°C for 24 h in unconscious adults resuscitated following out-of-hospital cardiac arrest with a shockable rhythm; for non-shockable rhythm and in-hospital cardiac arrest, temperature control was suggested as a weak recommendation [7].

One criticism of the original trials was that the temperature of the control groups in the two landmark studies [5,6] was not strictly normothermic but was slightly hyperthermic, around 37 to 38°C. This prompted a prospective randomized trial comparing strict normothermic control at 36°C with hypothermia at 33°C for 24 h (the targeted temperature management TTM1 trial) [8]. This trial published in 2013 showed no difference in mortality and neurological outcome between the two study groups. Consequently, the ERC guidelines in 2015 and 2021 extended the recommended post resuscitation target temperature to the wider range between 32 and 36°C [9,10].

In 2019, a RCT in patients after cardiac arrest with non-shockable rhythm showed improved neurological outcomes following treatment with hypothermia at 33°C compared to normothermia at 37°C [11]. In 2021, the further TTM2 randomized trial showed no difference in mortality and neurological outcome between hypothermia at 33°C and normothermia with early treatment of fever (body temperature ≥37.8°C) [12]. In the same year, a meta-analysis was published, concluding that in adults following cardiac arrest, the use of TTM in the range of 32 to 34°C, when compared to normothermia, did not result in improved outcomes [13]. Consequently, the latest ERC guidelines in cooperation with the European Society of Intensive Care Medicine (ESICM) recommended preventing fever in patients resuscitated from cardiac arrest, with an amended recommendation that there was insufficient evidence to recommend for or against temperature control at 32 to 36°C, but that some subgroups of patients may benefit from such temperature control [14].

## Critical appraisal of the current 2022 ERC/ESICM guidelines and new scientific evidence

There are a number of important limitations to the two large TTM studies [8,12], that have greatly affected the guidelines over the last few years. Firstly, the rate of bystander cardiopulmonary resuscitation in all groups was 73 to 82%, which is considerably higher than the average rate in Europe of 58% [1]. Observational data and comparative analysis show that patients with a short cardiac arrest time, as it is in the case of bystander CPR, presumably have less brain damage and so might not benefit from hypothermia, as the beneficial effect of hypothermia increases with a longer duration of cardiac arrest [15,16]. Secondly, both TTM studies allowed a delay of up to three to four hours between ROSC and randomization, and the targeted temperature has taken up to 7 h after cardiac arrest to achieve. Reperfusion injury, however, starts immediately following resuscitation from cardiac arrest, and all pathophysiology shows that earlier cooling is more effective. In previous randomized studies showing a benefit of hypothermia, cooling was initiated by the ambulance service [6] or after a median delay of 105 min [5]. Thirdly, both TTM studies included many centers from various countries, with each center enrolling only a few patients. This creates potential for considerable heterogeneity in all other aspects of post-resuscitation care. For this reason, a possible dose-response effect may not be detected at this level of heterogeneity.

The latest recommendations on temperature management by ERC/ESICM [14] are predominantly based on the meta-analysis by Granfeld *et al*. [13] In this meta-analysis [13], the selected studies were separated into two different analyses. One meta-analysis included only studies reporting outcome at discharge or 30 days, and the other included only studies reporting outcome at 3 months or 6 months. Both meta-analyses showed a risk ratio in favor of hypothermia at 32 to 34°C compared to normothermia, however, the 95% confidence interval crossed 1, and so the results of these two group analyses were not considered statistically significant. Splitting the analysis in two different outcome evaluation time points reduced the number of eligible studies and subsequently reduced the overall power of the studies in the meta-analysis, limiting ability to demonstrate a positive effect. There was no meta-analysis summarizing all available data on the underlying study question. Why the data was split into these underpowered groups is not clear. In addition, it was previously shown, that the proportion of good/poor outcome does not change over time [17], thus splitting the studies into different time points of outcome evaluation was not required, and performing one analysis of all studies may provide different results.

A number of retrospective studies demonstrated that a subgroup of patients with suspected moderate brain damage benefited the most from therapy with hypothermia in the range of 32 to 34°C. These are specifically the patient groups with a lower rate of basic life support [15], longer no-flow duration [16], intermediate duration from cardiac arrest to ROSC [18], higher lactate levels at arrival [19], moderate damage risk classification [20,21], and an EEG pattern suggesting moderate encephalopathy [22]. In total, these groups represent 40% and more of all included patients. All the results of these retrospective studies make pathophysiological sense, since a neuroprotective therapy may not be beneficial when the damage to the brain is too mild, or, on the other side of the range, too severe.

A Cochrane systematic review and meta-analysis on temperature management after cardiac arrest in adults has recently been published [23]. Due to their strict methodology, standardization, and transparency, Cochrane meta-analyses are considered to provide the highest level of evidence and quality [24]. This Cochrane meta-analysis represents the most recent and complete scientific evidence on temperature management after cardiac arrest, and includes 12 randomized trials. The authors found, that conventional cooling methods to induce therapeutic hypothermia in the range of 32 to 34°C compared to normothermia or no temperature control is associated with improved neurological outcomes after cardiac arrest [23]. The effect of hypothermia seemed to be highest in the subgroup with non-witnessed cardiac arrest, bystander CPR rates of less than 60%, no-flow times of more than one minute, and when hypothermia was initiated within two hours after ROSC [23]. One RCT in patients after in-hospital cardiac arrest, that showed no difference in neurological outcome between hypothermia in the range of 32 to 34°C and normothermia, was released after the Cochran systematic review was submitted to the editorial process [25]. However, the authors of the Cochrane meta-analysis have stated that pending formal assessment, it seems that including the result of this study [26] would not have changed the main conclusion [23]. Another recent meta-analysis confirms the beneficial overall effect of therapeutic hypothermia [27].

After publication of the very recent Cochrane review, there was another update of the review that served as basis of the 2022 ERC/ESICM guidelines [28]. The authors concluded that the updated meta-analysis showed no benefit from temperature control at 32 to 34°C compared with normothermia or 36°C, although the 95% confidence intervals cannot rule out a potential beneficial effect [28]. The Cochrane meta-analysis seems to be more complete since it included four additional RCTs, that were not included in the updated meta-analysis mentioned above.

## Summary of 2023 evidence

1. Animal studies with cardiac arrest models show a remarkable benefit from hypothermia in the range of 32 to 34°C on neuronal damage and neurologic outcome when hypothermia is induced early after ROSC.
2. Some RCT show a statistically significant benefit for hypothermia in the range of 32 to 34°C compared to normothermia or no temperature control after cardiac arrest, though other randomized controlled trials do not confirm this beneficial effect. Which patients may benefit from lower (32 to 34°C) or higher temperatures is still unknown.
3. Earlier and most recent meta-analyses of RCT show a statistically non-significant effect in favor of hypothermia in the range of 32 to 34° compared to normothermia or no temperature control in patients after cardiac arrest. In the most recent and comprehensive Cochrane systematic review and meta-analyses including all RCT, this beneficial effect of hypothermia in the range of 32 to 34°C compared to normothermia or no temperature control was statistically significant.
4. Several retrospective clinical studies indicate a beneficial effect of hypothermia in the range of 32 to 34°C compared to normothermia, especially in subgroups with presumable moderate brain damage.
5. There is no animal or human study showing that hypothermia in the range of 32 to 34°C compared to normothermia or no temperature control results in worse neurological or overall outcome.

## Recommendation 2023

Some uncertainty exists as to whether hypothermia in the range of 32 to 34°C compared to normothermia is beneficial in terms of improving neurologic outcome in all patients after cardiac arrest. The current recommendations from the ERC and ESICM to merely prevent fever, in our view, neither take into account all current available evidence, nor consider the shortcomings of studies. Based on retrospective studies showing that a large proportion of patients with presumable moderate brain damage significantly benefit from hypothermia in the range of 32 to 34°C, along with the most recent Cochrane systematic review and meta-analyses of RCT showing a statistically significant benefit of hypothermia in the range of 32 to 34°C, and based on the fact that no study has shown a deleterious effect of hypothermia in the range of 32 to 34°C on neurological or overall outcome, we suggest that international guidelines follow the current Cochrane analyses and in the interim period clinicians should consider hypothermia in the range of 32 to 34°C in all adult patients after cardiac arrest as soon as feasible, and to maintain this temperature range for at least 24 h. Active normothermia (36.5 to 37.7°C) should be ensured after rewarming before and during neuroprognostication to avoid fever.

Future randomized studies are needed to identify the patients who benefit most from hypothermia in the range of 32 to 34°C and to find the optimal time point of initiating and the optimal duration of hypothermia.

## Acknowledgements

### Conflicts of interest

Wilhelm Behringer: Speakers honoraria from Zoll Medical Corporation and Becton Dickinson GmbH. Bernd W. Böttiger: Speakers fees from Forum für medizinische Fortbildung (FomF), Baxalta Deutschland GmbH, ZOLL Medical Deutschland GmbH, C.R. Bard GmbH, GS Elektromedizinische Geräte G. Stemple GmbH, Novartis Pharma GmbH, Philips GmbH Market DACH, Bioscience Valuation BSV GmbH, Becton Dickinson GmbH, Fundacja Polski Instytut Evidence Based Medicine. Daniele G. Biasucci: Honoraria from Vygon SAS for having written educational materials for their website. Jim Connolly: Educational Honoraria from Sonosite; ultrasound machine placement from Terrason, Cannon, Echonous; Personal shares Smith & Nephew, GSK, Inovio, Linde, but does not have any conflicts of interest specifically related to the topic of the manuscript. Abdo Khoury: Speakers honoraria from Zoll Medical Corporation, Archeon Medical, Aguettant, Vygon, Baxter, Fisher Paykel, but does not have any conflicts of interest specifically related to the topic of the manuscript. Giuseppe Ristagno: Participated on the scientific advisory board of Philips Healthcare but does not have any conflicts of interest specifically related to the topic of the manuscript. For the remaining authors, there are no conflicts of interest.
